# Imaging of refractory chronic recurrent multifocal osteomyelitis succesfully treated with etanercept and methotrexate

**DOI:** 10.1186/1546-0096-12-S1-P166

**Published:** 2014-09-17

**Authors:** Estefania Quesada-Masachs, Carolina Díaz Mendoza, Ignacio Barber, Consuelo Modesto Caballero

**Affiliations:** 1 Pediatric Rheumatology, Hospital Universitari Vall d'Hebron, Barcelona, Spain; 2 Rheumatology, Hospital Universitari Vall d'Hebron, Barcelona, Spain; 3 Pediatric Radiology, Hospital Universitari Vall d'Hebron, Barcelona, Spain

## Introduction

Chronic recurrent multifocal osteomyelitis (CRMO) is a rare form of chronic nonbacterial osteomyelitis of unknown etiology and, to date, there is no uniformly effective treatment for this disease. Role of magnetic resonance imaging (MRI) to detect lesions in CRMO has increased last years.

## Objectives

To describe a patient affected by an atypical clinical form of CRMO refractory to treatment who finally responded to combination therapy with Etanercept (ETN) and Methotrexate (MTX).

## Methods

A 5-year-old boy presented with a 1 year history of right leg pain with limited range of motion and difficulty in walking due to flexion, abduction and external rotation of right hip attitude. No fever. Plain radiographs were normal. In the blood test, elevation of acute phase reactants was detected: ESR 41 mm/h, CRP 2.27 mg/dL. A Whole Body MRI (WB-MRI) scan revealed osteitic multifocal lesions with edema and enhanced T2 signal without necrosis in: proximal epiphysis and metaphysis of right hip, proximal metaphysis of left femur, left isquium, left acetabular bottom, right acetabulum, sacrum and proximal metaphysis of right tibia. Intense bilateral sacroiliitis and mild synovitis in right hip were also observed. All this findings were suggestive of CRMO. Synovium biopsy showed chronic synovitis. Bone biopsy of femoral neck lesion was anatomopathologically compatible with CRMO with signs of acute inflammation. Cultures of both samples and synovial fluid were negatives. No infection or malignancy was identified in the extended study confirming the diagnosis of CRMO.

## Results

He received several courses of intravenous and oral antibiotics without improvement. NSAID regimen was ineffective too. Intravenous Pamidronate was initiated adding oral prednisone and Methotrexate 15 mg/m2/week sc to treatment. Twelve months later he had improved pain and motion but persisted elevation of ESR and CRP, a slightly limitation of right hip rotation and he reported inflammatory back pain with 1 hour of daily morning stiffness. A new MRI scan of the spine was performed revealing inflammatory lesions in D10, D11 and D12 vertebral pedicles suggestive of multiple osteomyelitis foci. Etanercept 0.8 mg/kg/week was prescribed concomitantly with Methotrexate. One month after treatment initiation he reported improvement, without back pain, and six months later he remained symptom-free leading a normal life, with normal activity, without joint limitation of the hip during the physical examination and with normal acute phase reactants in blood tests. The last MRI performed to control the patient’s evolution revealed resolution of previous lesions without inflammatory osteo-articular activity.

## Conclusion

We suggest that anti-TNF therapies could be an effective treatment option in some patients with CRMO refractory to conventional treatment. Randomized controlled trials are needed to establish the role of these therapies in refractory CRMO. We also suggest that MRI is a useful tool in diagnosis and follow up of CRMO patients, providing a detailed description of the osteitis lesions and correlating the findings with the clinical evolution.

## Disclosure of interest

None declared.

